# The Effect of Dietary *Helianthus tuberosus* L. on the Populations of Pig Faecal Bacteria and the Prevalence of Skatole

**DOI:** 10.3390/ani10040693

**Published:** 2020-04-16

**Authors:** Monika Okrouhlá, Jaroslav Čítek, Roman Švejstil, Kateřina Zadinová, Kamila Pokorná, Daniela Urbanová, Roman Stupka

**Affiliations:** 1 Department of Animal Science, Faculty of Agrobiology, Food and Natural Resources, Czech University of Life Sciences Prague, 16500 Prague, Czech Republic; citek@af.czu.cz (J.Č.); zadinova@af.czu.cz (K.Z.); pokornakamila@af.czu.cz (K.P.); urbanovad@af.czu.cz (D.U.); stupka@af.czu.cz (R.S.); 2 Department of Microbiology, Nutrition and Dietetics, Faculty of Agrobiology, Food and Natural Resources, Czech University of Life Sciences Prague, 16500 Prague, Czech Republic; svejstil@af.czu.cz

**Keywords:** inulin, skatole, microbiota, *Escherichia coli*, entire male

## Abstract

**Simple Summary:**

The elimination of boar taint by a method other than surgical castration without anaesthesia is currently one of the main topics in pig research. Boar taint occurs in meat from some entire male pigs and is undesirable for sensitive consumers. Boar taint is mainly caused by skatole. Skatole is produced by the breakdown of proteins by intestinal bacteria and can be stored in meat and reduce its sensory quality (taste and odour). Boar taint can be reduced by a diet high in easily fermentable saccharides, such as Jerusalem artichoke (*Helianthus tuberosus* L.). These saccharides change the bacterial colonisation in the intestines and thus reduce the production of skatole. The aim of this study was to evaluate the effects of different levels of Jerusalem artichoke on performance, carcass composition and skatole and indole levels in adipose tissue and on microbiota in faecal samples. In the present study, Jerusalem artichoke had no negative effect on the growth performance or carcass value in male pigs. Moreover, Jerusalem artichoke led to decreased skatole levels in the adipose tissue, probably due to the decreased level of proteolytic bacteria, which cause a higher rate of skatole production in the gastrointestinal tract. It seems that a dietary concentration of 8.1% of Jerusalem artichoke fed 13 days before slaughter is a sufficient dose for decreasing the skatole levels to those of castrated males, and this approach could be an alternative to the surgical castration of male pigs.

**Abstract:**

Jerusalem artichoke contains inulin polysaccharide, which has prebiotic effects and influences the microbiota of the digestive tract. The addition of Jerusalem artichoke in boar diets may decrease the content of skatole and indole, which are the main constituents of boar taint, and may also negatively affect the taste and odor. The objective of this study was to evaluate the effects of different levels of *Helianthus tuberosus* L. (*H. tuberosus*) in feed mixtures on performance, carcass composition, the levels of microbiota in faecal samples, and the concentrations of skatole and indole in adipose tissue. The study was performed with 47 crossbred entire male pigs of the Large White _sire_ × (Large White _dame_ × Landrace) genotype fed a basal diet with 0%, 4.1%, 8.1% or 12.2% *H. tuberosus* for 13 days before slaughter. Significant differences in daily weight gain and daily feed intake were found (*p* = 0.045), with the values being lower in the group with the highest level of *H. tuberosus.* In addition, increasing levels of *H. tuberosus* decreased the concentration of skatole in the adipose tissue (*p* = 0.003). The highest level of *H. tuberosus* decreased the level of *Escherichia coli* (*p* ≤ 0.001) in the faeces. The enterococcal count increased (*p* = 0.029) in groups with a diet that included 4.1% and 8.1% *H. tuberosus.* There was also a significant correlation between the concentration of *H. tuberosus* and the concentration of *E. coli* (*p* < 0.001; −0.64) and the skatole levels in the adipose tissue (*p* = 0.001; –0.46). Moreover, there was also a positive correlation between the concentration of *E. coli* and the skatole levels in the adipose tissue (*p* = 0.023; 0.33). In conclusion, feeding pigs with *H. tuberosus* leads to decreased levels of skatole in the adipose tissue. According to the results of our study, a diet with 8.1% *H. tuberosus* is sufficient for decreasing skatole levels, which could be due to the decreased levels of pathogenic bacteria in the intestines.

## 1. Introduction

Boar taint and its elimination are among the problems associated with entire male pigs. At present, the European Union aims to ban surgical castration without anaesthesia. The use of lidocaine-based local analgesia is time consuming and costly and may also induce stress to the animals due to extra handling [1]. It is important to focus on other strategies to decrease boar taint. The high concentrations of some compounds cause distinctive boar taint in entire male pigs. Specifically, these compounds include androstenone, indole and skatole [2,3]. Androstenone is a steroid with an odour typical of urine. It is synthesised in testes and metabolised in the liver [4]. Skatole, which is formed by tryptophan degradation in anaerobic conditions, has a characteristic offensive faecal odour. It is produced in the gastrointestinal tract (GIT), where L-tryptophan is cleaved by *Escherichia coli,* clostridia and lactobacilli in the intestines. Most of these bacteria are able to metabolise tryptophan to indole and indole acetic acid, which is the main precursor of skatole. Indeed, only a small quantity of intestinal bacteria, i.e., less than 0.01%, is able to catalyse the decarboxylation of indole acetic acid to skatole [5]. Skatole is typically metabolised in the liver in two phases. In boars, there is insufficient metabolisation of androstenone and skatole in the liver; therefore, these substances accumulate in the adipose tissue [6]. Consumer sensitivity to boar taint depends on the individuality of the person. In general, women tend to be more sensitive to the boar taint than men [7].

To some extent, the production of skatole in the gastrointestinal tract can be influenced by nutrition. Skatole formation can be reduced when the diet of animals is supplemented with a high quantity of easily fermentable saccharides, which are not digested by enzymes in the small intestine [8]. These saccharides are prebiotics; thus, oligosaccharides support the activity and growth of Bifidobacteria and inhibit the growth of the bacteria involved in skatole and indole formation, e.g., *E. coli* and *Clostridium* spp. [9]. One of these oligosaccharides with prebiotic function is inulin, which passes in intact form through the upper to the lower parts of the gastrointestinal tract, where it undergoes bacterial fermentation and is able to change the microbial diversity [10]. The end products of bacterial fermentation are gases, such as carbon dioxide and hydrogen, lactate and short-chain fatty acids (i.e., acetate, propionate and butyrate). It is widely accepted that the presence of inulin is able to change the composition of microbiota in the colon in favour of specific bacterial groups, such as Bifidobacteria [9,11]. These changes in bacterial fermentation in the colon could result in the reduction of some potentially pathogenic bacteria and thus the reduction of skatole production [12].

Chicory root and *Helianthus tuberosus* (Jerusalem artichoke) are examples of inulin sources, and these plants have a high inulin content. Many studies have demonstrated that providing feed with chicory or pure inulin influenced the content of skatole in the excrement, blood and adipose tissue of animals [13,14,15]. Furthermore, previous studies have shown that diets with chicory roots, dried chicory or pure inulin significantly decreased skatole in adipose tissue to levels equivalent to those of castrated males [16,17,18]. According to some studies, feeding inulin to pigs could also have a beneficial influence on growth performance, especially on daily weight gain [19,20].

Many authors have monitored the effect of chicory root on the skatole levels in adipose tissue; however, there is limited information on the effect of *H. tuberosus*, which has a comparable inulin content to that of chicory and a similar effect on boar taint. Therefore, the objective of this study was to investigate the effect of different levels of *H. tuberosus* on growth performance, carcass quality, skatole and indole concentrations in adipose tissue and on microbiota composition.

## 2. Materials and Methods 

The feeding experiment was conducted at the Ploskov Test Station, the external workplace of the Department of Animal Husbandry of the Czech University of Life Sciences Prague, in the Czech Republic. The experiment was approved by the Ethics Committee of the Central Commission for Animal Welfare at the Ministry of Agriculture of the Czech Republic and was carried out in accordance with Directive 2010/63/EU for animal experiments. The local Ethics Commission, case number 02/2018, approved all the procedures described in this study.

### 2.1. Diet and Animals

In the experiment, a total of 72 crossbred entire male pigs of the Large White _sire_ × (Large White _dame_ × Landrace) (LW_S_ × (LW_D_ × L)) genotype were used. Two animals were housed in each pen, and 36 pens were used. The average initial weight was 46.6 kg, and the average slaughter weight was 112.1 kg. The pigs were separated into four dietary treatments, and each treatment received a different diet: a basal diet containing extracted soya bean, wheat and barley meal and feed additive (premix); the basal diet +4.1% *H. tuberosus*; the basal diet +8.1% *H. tuberosus*; or the basal diet +12.2% *H. tuberosus. H. tuberosus* used in the diets was dried and milled (particle size ≤ 2 mm). The content of pure inulin was determined based on the analysis of the dried *H. tuberosus.* The basal diet was formulated according to the nutrient needs of the animals and fed ad libitum. The chemical compositions of the diet and dried *H. tuberosus* are shown in Table 1. The animals had free access to water throughout the course of the experiment. The animals were fed the basal diet between 93 and 140 days old. This period was followed by a 13-day period (from 140 to 153 days old) before slaughter, during which the dried and milled *H. tuberosus* was homogenously mixed into the basal diet for each experimental group every day. The animals were slaughtered at the age of 153 days. The pigs were housed in pairs in pens (with concrete floor grates) designed for feeding (1 feeder for 2 pigs), and the average daily feed intake was observed. The average daily weight gain was observed by weighing the animals once a week. At the end of the experiment, all pigs were weighed. Based on an average body weight (average ± 5 kg), 11–13 pigs from each treatment were selected. All selected pigs were close to mean weight of each treatment. Selected pigs were slaughtered at a commercial slaughterhouse and subjected to analyses.

### 2.2. Sample Collection

For the microbiological analysis, 0.5 g of uncontaminated fresh faeces was collected from the rectum of each animal one day before slaughter. The samples were collected in sterile tubes with 9 mL of anaerobic solution containing nutrient broth and tryptone (Oxoid Ltd., Basingstoke, UK) in an oxygen-free environment developed by injecting carbon dioxide into the tube. The faecal samples were immediately processed for microbiological analysis. For the analyses of the skatole and indole, samples of adipose tissue were collected from the neck region 24 h post-mortem and frozen at −80 °C until the analyses. The hot carcass weight and lean meat percentage (i.e., using a two-point (ZP) method) were measured 45 min post-mortem at the slaughterhouse.

### 2.3. Skatole and Indole Analyses

The analysis of the skatole and indole concentrations in the adipose tissue was performed using HPLC (Jasco LC-2000, Watrex Praha, s.r.o., Prague, Czech Republic) based on a method described by [22].

For the skatole and indole determination, a Kinetex C18 100A (5 μm, 50 × 4.60 mm ID) column was used at a 40 °C operating temperature. The mobile phase parameters were as follows: A—potassium phosphate buffer (10 mM) and B—methanol. The gradient profile programme was as follows: 0–0.2 min, 90% A; 0.2–6.0 min, 90%–55% A; 6.0–7.0 min, 55%–0% A. The column flow was 1.2 mL/min, with an injection volume of 30 μL. Fluorescence detection was performed with excitation at 285 nm and emission at 340 nm. For the determination of skatole and indole from the sample, a standard calibration curve was used.

For skatole and indole content, the proportion of samples above the detection level was calculated.

### 2.4. Microbial Analysis

The plate count method was used to evaluate the composition of the faecal microbiota. The groups of bacteria tested are shown in Table 2. The total counts and bifidobacteria were cultivated at 37 °C for 48 h in anaerobic conditions using the AnaeroGen anaerobic generation system (Oxoid Ltd., Basingstoke, UK). The lactobacilli were cultivated under microaerophilic conditions using the double-layered plate method at 37 °C for 48 h. The enterococci were cultivated aerobically at 37 °C for 48 h, and the *E. coli* and coliform bacteria were cultivated aerobically at 37 °C for 24 h. The cultivation medium for each group of bacteria is shown in Table 2.

### 2.5. Statistical Analysis

One-way analysis of variance (ANOVA) with the *H. tuberosus* content in diet as the fixed factor was used. Live weight, carcass weight and pen had no significant effect on evaluated characteristics, and therefore they were not included in the final model. The data were evaluated using the general linear model (GLM) procedure in SAS (version 9.04, Statistical Analysis System, Toronto, ON, Canada). The significance of the variance between the groups was tested using the Scheffe test. The significance level was *p* ≤ 0.05 for all the measurements. A Pearson correlation analysis was used to test for correlations. Residuals were checked for normality and were distributed normally.

Testing of significant differences was carried out according to the following mathematical statistical one-way analysis model:

Yi = µ + di + ei(1)

where: 

Yi = value of the trait; 

µ = overall mean; 

di = effect of the *H. tuberosus* content in diet (i = 1, 2, 3, 4); 

ei = random residual.

## 3. Results and Discussion

### 3.1. Growth Performance and Carcass Traits

The effects of the *H. tuberosus* concentration in the diet on growth performance and carcass traits are presented in Table 3. With regard to growth performance, a significantly lower (*p* = 0.042) daily weight gain (DWG) was found in the group with the highest amount of *H. tuberosus* compared with that of the other groups. On the other hand, Houdijk et al. [23], who studied the use of fructooligosaccharides in feed and their influence on growth performance, did not find any significant differences between groups fed with or without non-digestible oligosaccharides. Nevertheless, these results are in contrast to studies by Grela et al. [19] and Wang et al. [24], who found significantly higher daily weight gains and lower feed conversion ratios in pigs fed a diet supplemented with inulin. The decrease in the DWG (*p* = 0.042) in the group fed 12.2% *H. tuberosus* could be explained by the decreased daily feed intake in this group, which may have been caused by the presence of substances that altered the organoleptic properties of the feed mixture.

The amount of *H. tuberosus* in the diet had no significant influence on carcass traits, which is in agreement with the results reported by Vhile et al. [25], who found no significant differences between groups fed with or without *H. tuberosus*.

### 3.2. Skatole and Indole Concentrations in the Adipose Tissue

The effects of the *H. tuberosus* concentration in the diet on the skatole and indole concentrations in the adipose tissue are presented in Table 4. No significant effect was found on the concentrations of indole. These results are in accordance with those of previous studies, which found no significant difference between animals fed with or without different types of carbohydrates [26,27,28,29].

With regard to the level of skatole, a significant decrease was found when diets containing 8.1% and 12.2% *H. tuberosus* were fed to the animals. These results are in agreement with those of several studies, in which the presence of non-digestible oligosaccharides in the diet decreased the levels of skatole in adipose tissue [14,15,16,18,30]. In addition, Vhile et al. [25] fed animals diets with different levels of *H. tuberosus* only 7 days before slaughter, and they determined that adding dried *H. tuberosus* resulted in a dose-dependent decrease in skatole levels in the adipose tissue.

### 3.3. Microbiota Concentration in the Rectum

Diet and the presence of probiotics, prebiotics or synbiotics can influence microbiota [31]. In the present study, no significant difference was found in the total counts of anaerobic bacteria, bifidobacteria, lactobacilli or coliforms (Table 5), which is in accordance with the findings of Böhmer et al. [31]. A significant effect of *H. tuberosus* was detected in the count of the enterococci (*p* = 0.029), which belong to the order Lactobacillus. The groups given diets supplemented with 4.1% and 8.1% *H. tuberosus* had higher enterococcal contents. Likewise, Paßlack et al. [32] found that inulin increased the cellular numbers of enterococci in the faeces of animals. On the other hand, some authors did not find an effect of inulin on the amounts of enterococci [33,34]. It seems that differences in the results could be caused by variation in the basal diets or the levels of inulin.

Probiotic bacteria are able to selectively ferment prebiotic saccharides and have a positive effect on the maintenance of balanced intestinal microbiota [35].

In the present study, there was a significant difference (*p* ≤ 0.001) in the level of *E. coli* with increasing levels of *H. tuberosus* in the diet. *E. coli* may be responsible for the transition of indole acetic acid to skatole [36]. The reduction in *E. coli* with an increasing amount of *H. tuberosus* in the diet could be explained by the antagonistic ability of probiotic bacteria to reduce the levels of enterobacteria, *E. coli* and other potentially pathogenic bacteria [37]. Many studies performed on piglets have shown that a diet enriched with inulin-type fructans has a positive effect on the gastrointestinal tract through an inhibition of pathogenic bacteria [38,39,40], a reduction of intestinal pH and an increase in villous height [41].

### 3.4. Correlations between the Levels of H. tuberosus, the Microbiota and the Concentrations of Skatole and Indole 

The correlations between the levels of *H. tuberosus*, the microbiota and the concentrations of skatole and indole are shown in Table 6. Table 7 shows the correlations between the concentrations of the microbiota and the skatole and indole levels in the adipose tissue. The results show that increasing levels of *H. tuberosus* in the diet are significantly related to decreasing levels of *E. coli* in the rectum (*p* ≤ 0.001; −0.64). 

With a decreased content of *E. coli*, the levels of skatole in the adipose tissue subsequently significantly decreased (*p* = 0.023; 0.33). These results are in accordance with the fact that *E. coli* belong to the bacteria involved in one part of skatole production in the hind gut [9]. The decreased levels of *E. coli* may be caused by the prebiotic effect of inulin from *H. tuberosus* and the antagonistic ability of probiotics to reduce levels of pathogenic bacteria. The effect of dietary *H. tuberosus* could be enhanced by combining it with fermented liquid feed, which leads to decreased levels of coliforms and better performance [42,43].

## 4. Conclusions

In conclusion, feeding animals diets with *H. tuberosus* leads to a decreased level of skatole in the adipose tissue. This could be due to the decreased level of proteolytic bacteria, which cause a greater production of skatole, in the gastrointestinal tract. The enrichment of the diet does not have any major negative effects on the growth performance or carcass value. It appears that a diet with 8.1% of *H. tuberosus* fed 13 days before slaughter is a sufficient dose for decreasing the skatole levels, in boars with carcass weight of 110 kg, to those of castrated males.

## Figures and Tables

**Table 1:** Analysed chemical composition of the basal diet and *H. tuberosus.*

Nutrient Composition	Basal Diet	*H. tuberosus*
Dry matter (%)	90.95	90.00
Crude protein (%)	14.18	8.57
Ether extract (%)	1.06	0.33
Crude fibre (%)	3.63	3.51
Crude ash (%)	4.58	7.47
N-free extract (%)	67.50	70.12
Fructans (%)	-	55.76
MEp (MJ/kg)	13.25	13.79

MEp: metabolizable energy for pigs [21].

**Table 2:** Cultivation media used.

Group Tested	Cultivation Medium
Total counts of anaerobic bacteria	Wilkins–Chalgren anaerobe agar with the addition of soy peptone (W + S)
Bifidobacteria	W+S supplemented with mupirocin (100 mg/L) and glacial acetic acid (1 mL/L)
Lactobacilli	Rogosa agar supplemented with glacial acetic acid (1.32 mL/L)
Enterococci	Slanetz–Bartley agar
*E. coli* and coliform bacteria	TBX agar

All media were purchased from Oxoid^®^ (Oxoid Ltd., Basingstoke, UK). TBX: tryptone bile x-glucuronide medium.

**Table 3:** The effect of the dietary *Helianthus tuberosus* L. concentrations on the growth performance and carcass traits of entire males.

Levels of Dietary *H. tuberosus*	Control (n = 11)	4.1% (n = 12)	8.1% (n = 11)	12.2% (n = 13)	SEM	*p*-Value
	Means	Means	Means	Means		
Live weight at 93 days (kg)	46.6	46.9	46.1	46.0	0.69	0.968
Live weight at 153 days (kg)	112.1	114.4	113.3	109.0	1.14	0.354
DWG (g)	1184.3 ^a^	1176.1 ^a^	1194.8 ^a^	1084.4 ^b^	16.14	0.042
DFI (kg)	2.65 ^a^	2.66 ^a^	2.66 ^a^	2.37 ^b^	0.015	0.045
FCR (kg/kg)	2.24	2.26	2.23	2.19	0.011	0.935
Carcass weight (kg)	86.5	86.2	86.3	83.1	1.02	0.581
Dressing yield (%)	77.2	75.4	76.2	76.2	0.51	0.677
Backfat thickness (mm)	13.8	13.5	14.3	13.5	0.36	0.844
Lean meat share (%)	68.2	67.3	68.7	63.5	0.76	0.053

DWG: daily weight gain; DFI: daily feed intake; FCR: feed conversion ratio; DWG, DFI and FCR were evaluated between 93 and 153 days of age; ^a, b^ Means with different superscripts differ significantly; SEM: standard error of the mean.

**Table 4:** The effects of dietary *H. tuberosus* concentrations on skatole and indole concentrations in the adipose tissue of entire males.

Levels of Dietary *H. tuberosus.*	Control (n = 11)	4.1% (n = 12)	8.1% (n = 11)	12.2% (n = 13)	SEM	*p*-Value
	Means ± SD	Means ± SD	Means ± SD	Means ± SD		
Skatole (µg/g)	0.148 ^a^ ± 0.0143	0.077 ^ab^ ± 0.0059	0.027 ^b^ ± 0.0022	0.041 ^b^ ± 0.0040	0.0130	0.003
Skatole detection > 0.03 µg/g (%)	72.72	75.00	36.36	53.84		
Indole (µg/g)	0.059 ± 0.0051	0.045 ± 0.0036	0.063 ± 0.0086	0.050 ± 0.0026	0.0008	0.839
Indole detection > 0.03 µg/g (%)	81.82	58.33	54.54	69.23		

^a, b^ Means with different superscripts differ significantly; SEM: standard error of the mean

**Table 5:** The effects of dietary *H. tuberosus* concentrations on the microbiota in the rectums of entire males.

Levels of Dietary *H. tuberosus*	Control (n = 11)	4.1% (n = 12)	8.1% (n = 11)	12.2% (n = 13)	SEM	*p*-Value
	Means ± SD	Means ± SD	Means ± SD	Means ± SD		
Total anaerobes (log CFU/g)	9.91 ± 0.229	9.90 ± 0.331	9.95 ± 0.227	9.82 ± 0.264	0.038	0.706
Bifidobacteria (log CFU/g)	8.12 ± 0.337	8.37 ± 0.261	8.29 ± 0.303	8.42 ± 0.427	0.051	0.177
Lactobacilli (log CFU/g)	9.11 ± 0.322	9.38 ± 0.386	9.22 ± 0.392	9.19 ± 0.457	0.057	0.404
Enterococci (log CFU/g)	8.72 ^b^ ± 0.379	9.06 ^a^ ± 0.330	9.00 ^a^ ± 0.332	8.70 ^b^ ± 0.373	0.055	0.029
*Escherichia coli* (log CFU/g)	6.78 ^a^ ± 0.617	6.59 ^a^ ± 0.272	6.44 ^a^ ± 0.396	5.67 ^b^ ± 0.549	0.093	≤0.001
Coliforms (log CFU/g)	5.62 ± 0.539	5.45 ± 0.376	5.77 ± 0.828	5.52 ± 0.495	0.083	0.577

^a, b^ Means with different superscripts differ significantly; SEM: standard error of the mean; CFU: colony forming units.

**Table 6:** Pearson’s correlation coefficients between increasing dietary *H. tuberosus* and the concentrations of microbiota in the rectums and the levels of skatole and indole in the adipose tissue of entire males.

	Total Content	Microbiota in Rectums					Adipose Tissue	
		Bif.	Lactob.	Ent.	EC	Coli.	Skat.	Ind.
*H. tuberosus* concentration	−0.10	0.26	0.01	−0.06	−0.64 ^1^	−0.00	−0.46 ^2^	−0.02

Bif.: Bifidobacteria; Lactob.: Lactobacilli; Ent.: Enterococci; EC: *E. coli*; Coli.: Coliforms; Skat.: Skatole; Ind.: Indole; ^1^ *p* ≤ 0.001, ^2^ *p* = 0.001.

**Table 7:** Pearson’s correlation coefficient between the concentrations of microbiota in the rectums and the levels of skatole and indole in the adipose tissue of entire males.

	Skatole	Indole
Total anaerobes	0.11	0.11
Bifidobacteria	−0.15	0.04
Lactobacilli	−0.17	0.02
Enterococci	−0.16	0.05
*Escherichia coli*	0.33 ^1^	0.10
Coliforms	0.09	0.07

^1^*p* = 0.023.
